# RIF1 promotes human epithelial ovarian cancer growth and progression via activating human telomerase reverse transcriptase expression

**DOI:** 10.1186/s13046-018-0854-8

**Published:** 2018-08-03

**Authors:** Yong-Bin Liu, Ying Mei, Jing Long, Yu Zhang, Dong-Li Hu, Hong-Hao Zhou

**Affiliations:** 1 Department of Clinical Pharmacology, Xiangya Hospital, Central South University, 110 Xiang Ya Road, Changsha, 410008 People’s Republic of China; 2 Institute of Clinical Pharmacology, Central South University, Hunan Key Laboratory of Pharmacogenetics, Changsha, 410078 People’s Republic of China; 3 Department of Obstetrics & Gynecology, Xiangya Hospital, Central South University, Changsha, 410008 People’s Republic of China

**Keywords:** Ovarian cancer, RIF1, Telomerase reverse transcriptase, hTERT

## Abstract

**Background:**

Human telomerase reverse transcriptase (hTERT) is highly expressed in over 80% of tumors, including human epithelial ovarian cancer (EOC). However, the mechanisms through which hTERT is up-regulated in EOC and promotes tumor progression remain unclear. The aim of this study is to identify RIF1 as a novel molecular target that modulate hTERT signaling and EOC growth.

**Methods:**

RIF1 expression in ovarian cancer, benign and normal ovarian tissues was examined by immunohistochemistry. The biological role of RIF1 was revealed by MTS, colony formation and sphere formation assays. Luciferase reporter assay and chromatin immunoprecipitation (CHIP) assay were used to verify RIF1 as a novel hTERT promoter-binding protein in EOC cells. The role of RIF1 on tumorigenesis in vivo was detected by the xenograft model.

**Results:**

RIF1 expression is upregulated in EOC tissues and is closely correlated with FIGO stage and prognosis of EOC patients. Functionally, RIF1 knockdown suppressed the expression and promoter activity of hTERT and consequently inhibited the growth and CSC-like traits of EOC cells. RIF1 knockdown also inhibited tumorigenesis in xenograft model. RIF1 overexpression had the opposite effect. Luciferase reporter assay and ChIP assay verified RIF1 directly bound to hTERT promoter to upregulate its expression. The rescue experiments suggested hTERT overexpression rescued the inhibition of EOC cell growth and CSC-like traits mediated by RIF1 knockdown. Consistently, hTERT knockdown abrogated the RIF1-induced promotion of EOC cell growth and CSC-like traits.

**Conclusions:**

RIF1 promotes EOC progression by activating hTERT and the RIF1/hTERT pathway may be a potential therapeutic target for EOC patients.

**Electronic supplementary material:**

The online version of this article (10.1186/s13046-018-0854-8) contains supplementary material, which is available to authorized users.

## Background

Ovarian cancer is one of the most common gynecological malignancies throughout the world [1]. There are five types of ovarian cancer, among which epithelial ovarian cancer (EOC) accounts for about 90% of all reported cases and is the most lethal type [2]. Despite advances in therapeutic strategies, the overall prognosis of patients with EOC remains poor [3]. Therefore, it is urgent to better understand the molecular mechanisms in EOC tumorigenesis and progression and to discover new therapeutic targets for EOC.

Telomeres are repetitive DNA sequences at the ends of chromosomes and shorten with cell division, thereby limiting cell proliferation and serves as a significant barrier to prevent cancer initiation [4]. Human telomerase is a ribonucleoprotein enzyme complex which is composed of an RNA template (hTERC) and the telomerase reverse transcriptase (hTERT) for telomeric DNA synthesis [5]. hTERT, the rate-limiting catalytic subunit of telomerase, positively regulates telomerase activity at the transcriptional level. Particularly, hTERT is overexpressed in over 95% of ovarian cancers compared with normal epithelial cells [6]. In addition to stabilizing telomere length, hTERT has been demonstrated to be a central regulator of multiple hallmarks of cancer, including proliferation, survival and cancer stem cells (CSC) traits [7–9]. CSCs including the ovarian cancer stem cells play crucial roles in tumor promotion, recurrence, metastasis and other malignant characteristics of human cancers [10–12]. Therefore, hTERT is a potential biomarker and therapeutic target for EOC. Nevertheless, it is still unclear why hTERT expression is low in normal cells but upregulated during the process of carcinogenesis.

RIF1 (replication timing regulatory factor 1) was originally identified in budding yeast as RAP1 interacting factor 1 with a prominent role in telomere length regulation [13]. RAP1 is a member of the shelterin complex which play a fundamental role in the protection of telomeres. However, the structure and function of RIF1 are not conserved from yeasts to mammalian cells. In mammalian cells, RIF1 modulates pathway choice in DNA double-strand break (DSB) repair as well as replication timing regulation [14–17]. RIF1 is also involved in embryonic stem cells (ESCs) self-renewal and highly expressed in mouse embryonic stem cells [18–20]. In the field of cancer research, RIF1 was reported upregulated in breast cancer tissues and we previously found that RIF1 knockdown decreased cell growth and increased cisplatin sensitivity of cervical cancer cells [21, 22]. However, the specific role of RIF1 in EOC remains little known. In this study, we show that RIF1 expression is upregulated in EOC tissues and is closely correlated with clinical stage and prognosis of EOC patients. Moreover, we have discovered and identified that RIF1 has a novel molecular feature in binding at the promoter of *hTERT* in EOC cell lines by chromatin immunoprecipitation assay and luciferase reporter assay. Furthermore, the binding of RIF1 at the *hTERT* promoter activated hTERT expression in EOC cells, thereby promoting EOC cell growth and CSC-like traits. The rescue experiments suggested hTERT overexpression rescued the inhibition of EOC cell growth and CSC-like traits mediated by RIF1 knockdown. Consistently, hTERT knockdown abrogated the promotion of cell growth and CSC-like traits mediated by RIF1 overexpression in EOC cells. The results were confirmed by an in vivo nude mouse xenograft model. In summary, our results suggested that RIF1 regulated EOC cell growth and CSC-like traits through the activation of hTERT, and demonstrated that the RIF1/hTERT signaling pathway could serve as a potential therapeutic target for EOC.

## Methods

### Patients and samples

Ovarian cancer tissues, ovarian benign tumor tissues and noncancerous epithelial tissues from 104 patients who underwent surgical resection were obtained from Xiangya Hospital of Central South University (Changsha, Hunan, China) and Hunan Cancer Hospital (Changsha, Hunan, China) from 2010 to 2015. Written informed consent was obtained from all patients and this study was approved by the Ethics Committee of Xiangya School of Medicine, Central South University (Registration number: CTXY-140002-10). After fixation in 10% formalin, the collected tissues were embedded in paraffin for histological diagnosis and immunohistochemical staining. All other demographic and clinical information were acquired from the 2 hospitals mentioned above. Bioinformatic data was obtained from the human protein atlas (www.proteinatlas.org), Oncomine database (www.oncomine.org), Kaplan-Meier plotter database (http://kmplot.com/analysis/) and TCGA database.

### Immunohistochemistry

All tissue specimens were collected via biopsy of paraffin-embedded samples for immunohistochemistry (IHC) analysis in the Pathology Department of Xiangya Hospital or Hunan Provincial Tumor Hospital. Tissue sections (4 μm thick) were cut from paraffin embedded blocks. The tumor sections on slides were baked at 60 °C for 30 min followed by incubation in xylene for 3 × 10 min and rehydration through graded ethanol to distilled water. Antigen retrieval was done by heating samples in 1 mmol/L EDTA for 20 min. Nonspecific staining was blocked by 10% goat serum in PBS buffer for 20 min at room temperature. The endogenous peroxidase activity was quenched by incubation in 3% H_2_O_2_ for 10 min. And then the slides were incubated with rabbit polyclonal monospecific RIF1 antibody or PBS control at 4 °C overnight followed by incubation with biotinylated goat anti-rabbit antibody and peroxidase-conjugated streptavidin. The 3,3′-diaminobenzidine tetrahydrochloride substrate kit (Zhongshan Goldenbridge) was used to visualize staining according to the manufacturer’s instructions and the hematoxylin and eosin were used to counterstain all samples before viewing with a Leica DMI 4000B inverted microscope.

All ovarian cancer tissue sections were reviewed by two experienced pathologists and staining of RIF1 was independently scored by two pathologists blinded to the clinical data using the semiquantitative immunoreactive score (IRS) system. The score of the RIF1 staining intensity were performed as previously described. [23] The percentage of RIF1-positive cells was scored as follows: < 25% = 1, ≥ 25 to 50% = 2, ≥ 50 to 75% = 3, and ≥ 75 to 100% = 4. The staining intensity was scored as follows: negative = 0, weak = 1, moderate = 2, and strong = 3. A final IRS score was then calculated by multiplying these two scores. The cut-off score was set to 4.0 according to receiver operating characteristic curves (ROC) analysis. If the final score was < 4, the tumor was considered to have low RIF1 expression; whereas the score ≥ 4 indicated high RIF1 expression.

### Cell culture and cell lines

OVCAR3 and A2780 cell line was purchased from the Institute of Biochemistry and Cell Biology, Chinese Academy of Sciences (Shanghai, China). These cells were cultured in RPMI-1640 media, supplemented with 10% fetal bovine serum (FBS) at 37 °C under an atmosphere of 95% air and 5% CO_2_.

### Plasmids, shRNA transfection and generation of stable cell lines

Two shRNA sequences targeting human RIF1 cDNA were synthesized by GenePharma: RIF1 KD1: 5’-GCCUUUGAGUUCCAUCCAUTT-3′, KD2: 5’-GGUCAUACCUUUAGUGGUUTT-3′. The targeting sequence of scrambled control of shRNA was 5’-UUCUCCGAACGUGUCACGUTT-3′. The shRNA sequence targeting human TERT cDNA was synthesized by GenePharma: hTERT KD: 5’-CCGAAGAAGCCACCUCUUUTT-3′, Lentiviral vectors pGLV3/H1/GFP were acquired from GenePharma. The sequence of 5’-TTCTCCGAACGTGTCACGT-3′ was used to generate lentivirus expressing shRNA targeting human RIF1, viral infection was performed according to the direction for use provided by GenePharma. RIF1 stable knockdown OVCAR3 cell lines were screened out over 15 days with 2 μg/ml puromycin. To verify the stable knockdown of RIF1 in these cell lines, RT-qPCR and western blot analysis were performed after 30 generations of cell culture. Full length human RIF1 expression construct, wild type and mutant hTERT promoter luciferase reporter plasmids were purchased from Sangon Biotech (Shanghai, China). Full length human TERT (#51637) expression construct was purchased from Addgene.

### Animal studies

All animal care and experimental procedures were performed according to the National Institutes of Health’s Guide for the Use and Care of Laboratory Animals, and were approved by the Institutional Review Board of Central South University (Changsha, China). Female BALB/c nude mice (4 weeks of age, 18–22 g) were obtained from Shanghai SIPPR - B&K Laboratory Animal Corp. Ltd., and fed under specific pathogen-free conditions at Department of Laboratory Animals, Central South University. To initiate tumors, 2 × 10^6^ control and RIF1 stable knockdown OVCAR3 cells were inoculated subcutaneously into each flanks of nude mice. The tumors were examined every 4 days, maximum (L) and minimum (W) length of the tumor were measured with calipers, and the tumor volumes were calculated as 0.5LW^2^. On day 24, the animals were euthanized and the tumors were excised and weighed.

### Western blotting

Western blotting was carried out as described previously [24]. In brief, for Western blotting, proteins were extracted using RIPA buffer mixed with protease inhibitors (1:100), phenylmethylsulfonylfluoride (PMSF, 1:100) and dithiothreitol (DTT, 1:100) at 4 °C for 30 min. The lysate was centrifuged at 13000 rpm at 4 °C for 15 min. The supernatants were collected and protein concentrations were measured using the BCA method. For Western blot analysis, protein samples were separated by 8% SDS-PAGE and transferred onto polyvinylidene difluoride membranes (Bio-Rad). The membranes were then blocked with 5% nonfat milk and incubated with primary antibodies overnight followed by washing and reaction with horseradish peroxidase–conjugated secondary antibody for 1 h at 37 °C. The reaction was detected by using ECL reagents (GE Healthcare) and the signals were captured by X-ray films. Antibodies against RIF1 (sc-515,573) and β-actin (sc-58,673) were obtained from Santa Cruz Biotechnology. Antibodies against NANOG (ab21624) and SOX2 (ab97959) were purchased from Abcam. Antibodies against PI3K (4257 T), p-PI3K (4228 T), AKT (4691 T) and p-AKT (4060 T) were purchased from Cell Signaling Technology. Antibody to hTERT (AP1410d) was purchased from Abgent.

### RNA isolation and real-time quantitative PCR

Total RNA was isolated from cultured cells or collected tissue samples by TRIzol reagent (Invitrogen) Reverse transcription was performed using PrimeScript RT reagent Kit With gDNA Eraser (TaKaRa), and the quantitative RT-PCR was performed using SYBR Premix DimerEraser (Perfect Real Time) kit (TaKaRa Bio Inc) on the Roche LightCycler480 (Roche). Sequences of primers are shown in Additional file 1: Table S1. Data were analyzed using the -2^ΔΔct^ method and the expression of β-actin was used as normalization control.

### MTS assay

For the cell viability assay, RIF1 knockdown or overexpressed cells were seeded in 96-well plates at a density of 3 × 10^4^ cells in 100 μl medium for 1 to 4 days. Cell viability was detected by MTS approach according to the protocol for Cell Titer 96 Aqueous-One-Solution Cell Proliferation Assay kit (MTS).

### Clone formation assay

RIF1 knockdown or overexpressed cells were seeded in 6-well plates at a density of 500 cells in 2 ml medium for 8 days. The colonies were then stained with 1% crystal violet and counted. All experiments were performed with 3 independent trials.

### Sphere formation assay

For sphere formation assay, 2 × 10^3^ cells were seeded in 24-well ultra-low cluster plates (Corning) for 9 days. Spheres were cultured in DMEM/F12 serum-free medium (Hyclone) supplemented with B27 (Gibco), 40 ng/μl EGF (Gibco), 10 ng/μl 1 bFGF (Peprotech).

### Luciferase reporter assay

A fragment containing the core promoter region of hTERT (− 1998 − + 96) was inserted between the XhoI and HindІІІ sites of the firefly luciferase vector pGL3-Basic (Promega, Madison, WI). The wild type and mutant hTERT promoter luciferase reporter plasmids were constructed by Sangon Biotech (Shanghai, China). Renilla luciferase control reporter vector pRL-TK was used as a control. Cells were seeded in 24-well plates (Corning) in triplicate. The indicated plasmids were transfected into the cells using FuGENE HD Transfection Reagent (Promega). 24 h after transfection, dual-luciferase reporter assays were performed according to the protocol by using a Dual-Luciferase Reporter Assay System (Promega) on a luminometer (Berthold).

### Chromatin immunoprecipitation (ChIP) assay

ChIP assay was performed using ChIP Kit (Bes5001, BersinBio, Guangzhou, China) according to manufacturer’s instructions. Briefly, the cells were fixed with 1% formaldehyde, and the cross-linking was quenched by adding in 100 μl of 1.375 M glycine per milliliter of culture. The samples were sonicated on ice to shear the DNA into 200 to 600 bp fragments. For each total cell lysate, one third was used as the DNA input control, another third was immunoprecipitated with anti-RIF1 antibodies, and the last third was subjected to non-immune rabbit IgG (Cell Signaling Technology). DNA fragments were purified by spin columns (Qiagen, Hilden, Germany), and Real Time PCR was performed to amplify the segment in the promoter region of hTERT with the following primers:

Prime 1, Forward: 5’-TCAAGTCACACCCACTGGTAAG-3′, Reverse: 5’-ATGGGATAACAGGTGGTCACAG-3′. Prime 2, Forward: 5’-ACTGCTGGTACTGAATCCACTG-3′, Reverse: 5’-GCCTGTAATCCCAGCCAAATG-3′. Prime 3, Forward: 5’-GTTTCCTCGCCATGCACATG-3′, Reverse: 5’-GGGGCGGTTTGGAAAATTTG-3′. Prime 4, Forward: 5’-ATTTCCTCCGGCAGTTTCTG-3′, Reverse: 5’-TTGGATCTAAGGGGCGAGAAAC-3′. Prime 5, Forward: 5’-TCCATTTCCCACCCTTTCTCG-3′, Reverse: 5’-AGGCCCGTCATTTCTCTTTG-3′.

### Statistical analysis

All data were described as mean ± standard deviation (SD). Two-tailed Student’s t test was used to derive the significance between two groups for continuous variables. A χ^2^ test or Fisher’s exact test was applied for qualitative variables. Survival curves were graphed using the Kaplan–Meier method, and the groups were compared using log-rank test. Statistical analysis was conducted using SPSS 18.0 (SPSS Inc.). *P* ≤ 0.05 was considered statistically significant.

## Results

### RIF1 is significantly overexpressed in EOC and positively correlates with poor prognosis in EOC patients

**Fig. 1:**
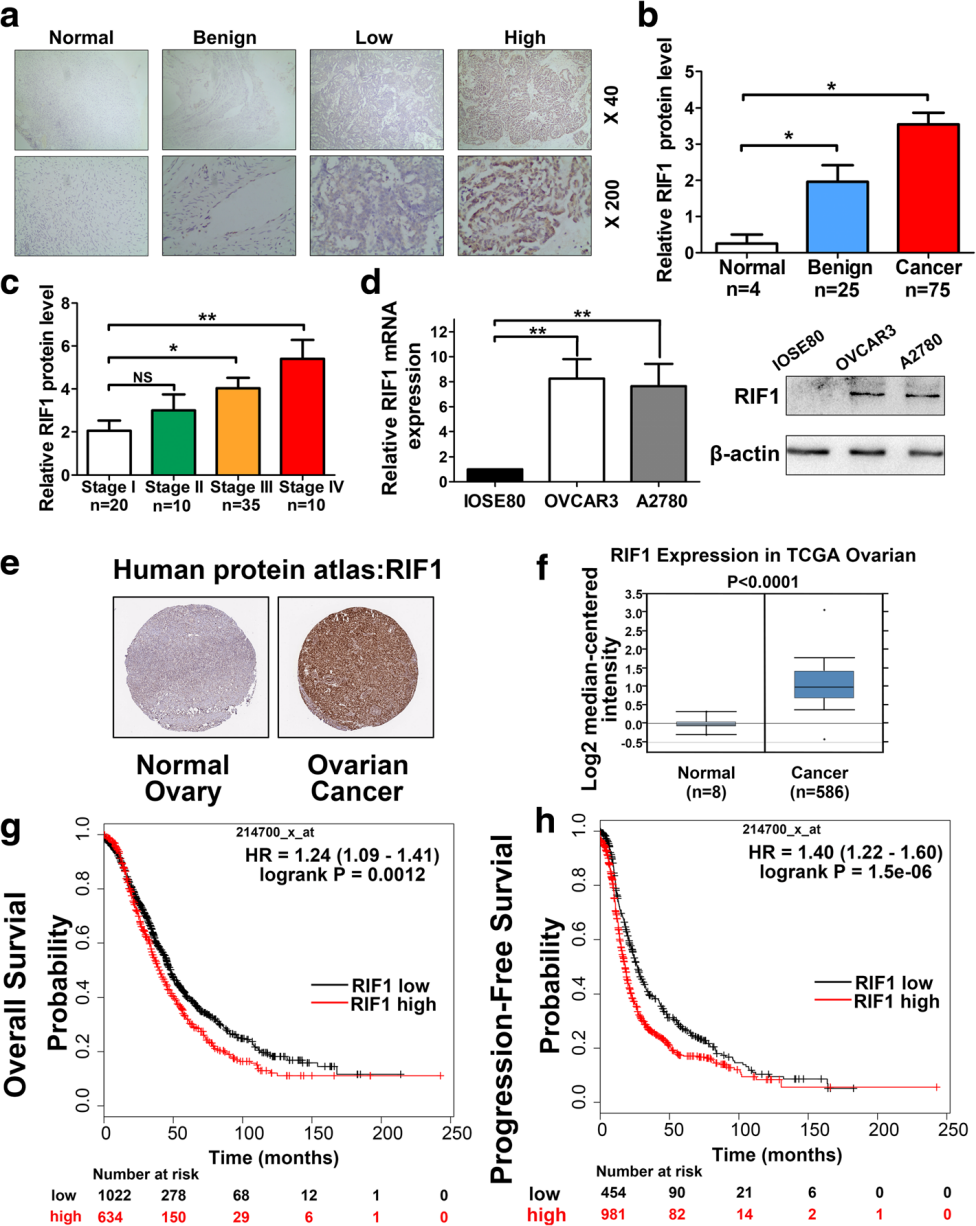
Clinical significance of RIF1 in human ovarian cancer patients. **a** Representative images of immunohistochemistry staining of RIF1 in specimens of EOC, normal and benign ovarian tissues. **b** Expression level of RIF1 in ovarian cancer progression was detected by IHC. **c** The expression of RIF1 was examined by IHC in different clinical stages of EOC tissues. **d** The mRNA and protein expression levels of RIF1 in ovarian epithelial cell line (IOSE80) and two human ovarian cancer cell lines (OVCAR3 and A2780) cell lines were detected by RT-qPCR and Western blot. **e** RIF1 protein expression in normal ovarian tissue and ovarian cancer specimens. Images were taken from the Human Protein Atlas online database. **f** RIF1 expression is overexpressed in ovarian cancer tissues compared with normal ovarian tissues. The identified and normalized data were taken from Oncomine database. **g, h** Kaplan-Meier analysis of overall survival and progression-free survival by low or high RIF1 expression in ovarian cancer patients were performed by using Cox proportional hazard models and follow-up data for 20 years. The data were obtained from Kaplan-Meier plotter database and all plots were analyzed by combing 15 data sets (GSE14764: *n* = 80, GSE15622: *n* = 35, GSE18520: *n* = 63, GSE19829: *n* = 28, GSE23554: n = 28, GSE26193: *n* = 107, GSE26712: *n* = 195, GSE27651: *n* = 49, GSE30161: *n* = 58, GSE3149: *n* = 116, GSE51373: n = 28, GSE63885: *n* = 101, GSE65986: *n* = 55, GSE9891: *n* = 285 and TCGA: *n* = 565). HR = hazard ratio. * *P* < 0.05, ** *P* < 0.01

To investigate the clinical relevance of RIF1 expression in ovarian cancer patients, we first examined RIF1 protein expression in ovarian cancer tissues, benign tissues and normal ovarian tissues by immunohistochemistry. Significantly higher expression levels of RIF1 were observed in ovarian cancer tissues compared with benign and normal ovarian tissues (Fig. 1a and b). We also observed that the RIF1 expression level was significantly associated with clinical stage (Fig. 1c, Additional file 2: Table S2). And then, we detected the mRNA and protein expression levels of RIF1 in normal ovarian epithelial cell line (IOSE80) and two human epithelial ovarian cancer cell lines (OVCAR3 and A2780). We found that RIF1 expression level in ovarian cancer cell lines were obviously higher than the normal ovarian epithelial cell line (Fig. 1d). Data from online databases were analyzed to confirm our results. We first analyzed the RIF1 protein expression in clinical tissue specimens from the human protein atlas (www.proteinatlas.org). We found that RIF1 had a positive strong expression in ovarian cancer, and negative weak staining in normal ovary (Fig. 1e). Consistently, in the Oncomine database, RIF1 mRNA level was higher in ovarian cancer tissues than that in normal ovarian tissues (*P* < 0.001) (Fig. 1f). Kaplan–Meier survival curves and log-rank test survival analysis demonstrated that overexpression of RIF1 mRNA was associated with worse overall survival (RIF1 low vs high expression patients: hazard ratio [HR] of survival =1.24, 95% confidence interval [CI] =1.09 to 1.41, *P* = 0.0012) (Fig. 1g) and progression-free survival of ovarian cancer patients (RIF1 low vs high expression patients: hazard ratio [HR] of survival = 1.4, 95% confidence interval [CI] = 1.22 to 1.6, *P* < 0.0001) (Fig. 1h). These results indicate that the expression level of RIF1 is upregulated in ovarian cancer tissues and aberrant activation of RIF1 is associated with poor prognosis of ovarian cancer patients.

### RIF1 promotes the growth of EOC cells in vitro

**Fig. 2:**
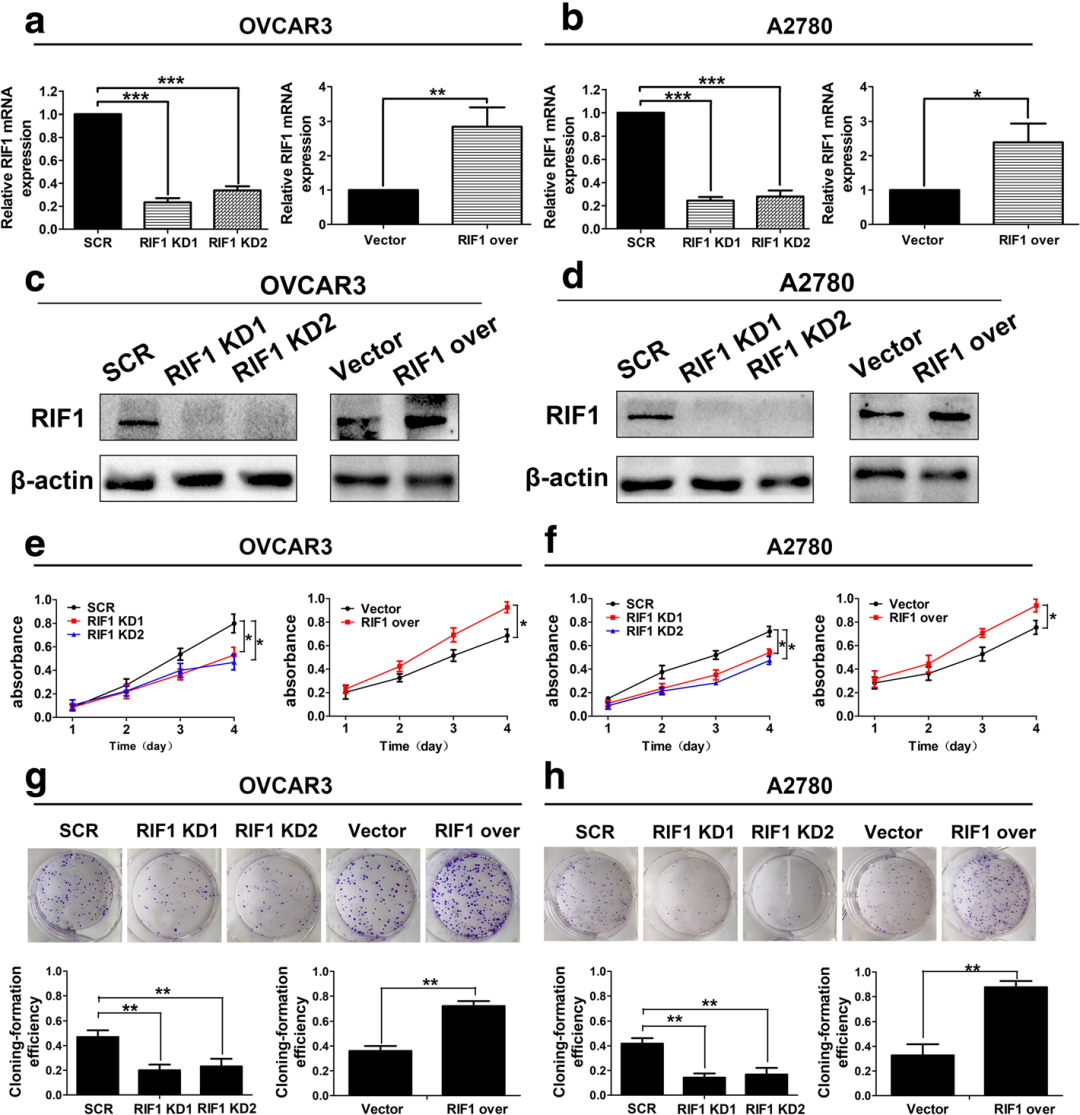
RIF1 promotes EOC cell growth in vitro. **a-d** Efficiency of RIF1 knockdown (RIF1 KD) by two shRNAs and RIF1 overexpression (RIF1 over) in OVCAR3 and A2780 cells was verified by real-time qPCR (**a** and **b**) and western blot (**c** and **d**). **e** and **f** Influence of RIF1 knockdown and overexpression on the cell viability of OVCAR3 (**e**) and A2780 (**f**) cells measured by the MTS assay. **g** and **h** Effect of RIF1 knockdown and overexpression on colony formation was measured in OVCAR3 and A2780 cells. Data were presented as mean ± SD of three independent experiments. * *P* < 0.05, ** *P* < 0.01, *** *P* < 0.001

RIF1 was knockdown by two shRNAs (RIF1 KD1 and RIF1 KD2) and overexpressed by full-length human RIF1 plasmid (RIF1 over) in two EOC cell lines. RIF1 expression was reduced about 80% of the scrambled control (SCR) and RIF1 expression in RIF1-overexperessed OVCAR3 and A2780 cells was increased to about 2.5 times compared with the vector control cells (Vector) (Fig. 2a-d). Knockdown of RIF1 significantly decreased the cell growth rate in both cell lines, whereas overexpression of RIF1 significantly increased the cell growth rate in both cell lines (Fig. 2e and f). In addition, RIF1 knockdown significantly decreased the numbers of OVCAR3 and A2780 colonies formed after culture compared with controls, whereas overexpression of RIF1 significantly increased the numbers of OVCAR3 and A2780 colonies formation in comparison with control cells (Fig. 2g and h).

### RIF1 promotes CSC-like traits of EOC cells

**Fig. 3:**
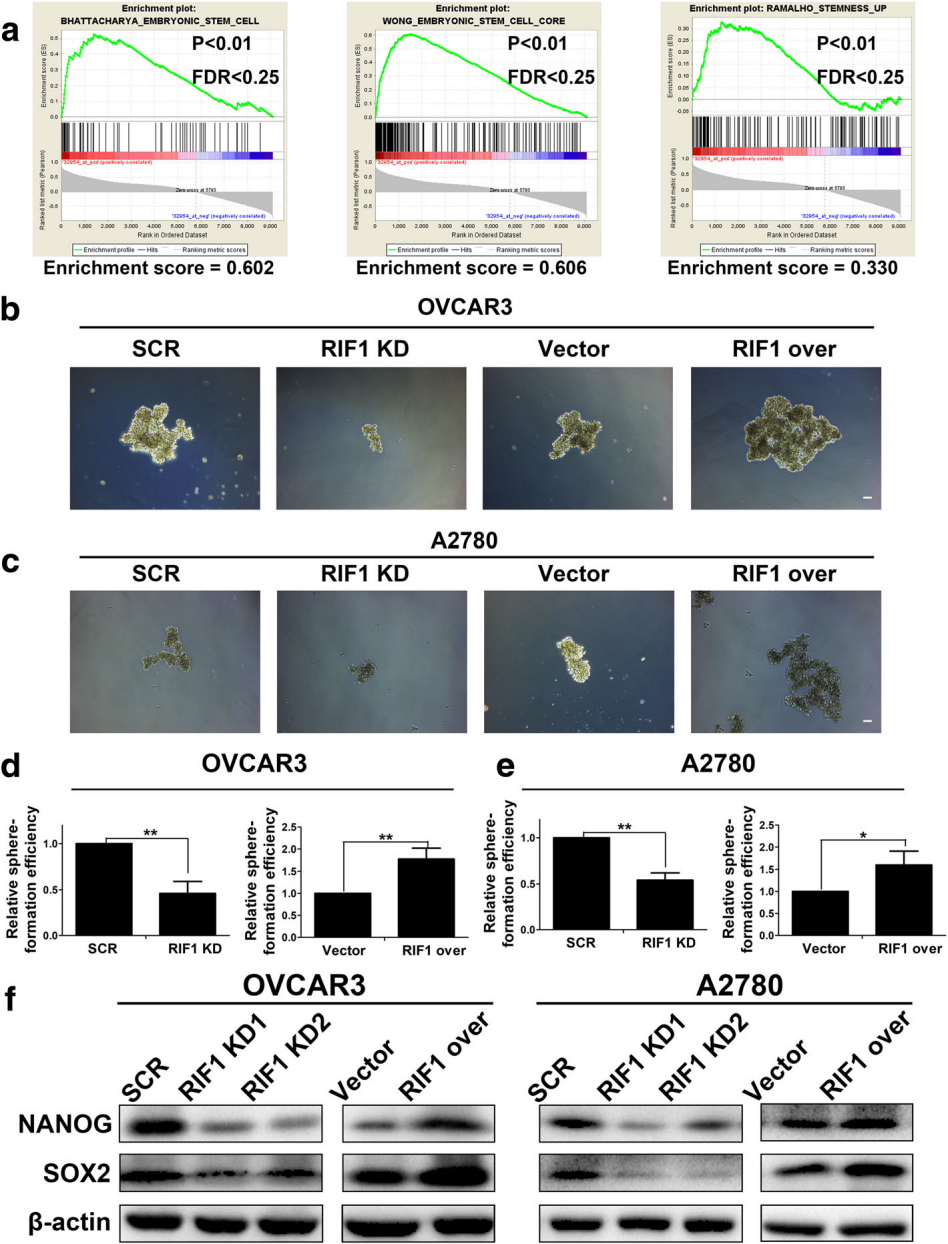
RIF1 enhances cancer stem cell-like properties in vitro. **a** GSEA plot showing RIF1 expression level was positively correlated with activated stemness-related gene signatures in the GEO data set. (NCBI/GEO/GSE7463; *n* = 43). **b** and **c** Representative images of spheres formed by the OVCAR3 and A2780 cells. Scale bar, 100 μm. **d** and **e** Quantification of sphere-formation efficiency of the indicated cells. **f** Western blot analysis of the protein expression of the stemness-associated markers including NANOG and SOX2 in RIF1-silenced and overexpressed OVCAR3 and A2780 cells. Date were shown as mean ± SD of three independent experiments. * *P* < 0.05, ** *P* < 0.01

Since RIF1 is reported to be involved in ES self-renewal and is highly expressed in mouse embryonic stem cells (ESCs) [18], we explored whether the expression of RIF1 is related to cancer stem cell-like (CSC-like) traits in EOC cells. Gene set enrichment analysis (GSEA) indicated that RIF1 expression closely correlated with the expression of a set of stemness regulating genes in the publicly available ovarian cancer patient expression profiles (GSE7463; Fig. 3a). We then investigated the effect of RIF1 on CSC-like properties by sphere formation assay. When OVCAR3 and A2780 cells were dissociated into single cells, seeded into non-adhesive coating culture plates and cultured with specific medium, spheres appeared to contain enriched CSC population [25]. The size and number of spheres were decreased about 50% in RIF1-silenced OVCAR3 and A2780 cells whereas RIF1-overexperessed OVCAR3 and A2780 cells formed significantly larger and more spheres than vector control cells (Fig. 3b-e). In addition, the protein expression levels of the genes related to cancer stemness, including NANOG and SOX2 [26, 27] were significantly downregulated in RIF1-knockdown EOC cells and were upregulated in RIF1-overexpressed EOC cells (Fig. 3f). Taken together, our data indicated that RIF1 promoted cancer stem cell-like properties of EOC cells.

### Clinical relevance of the expression of RIF1 and hTERT in EOC tissues

**Fig. 4:**
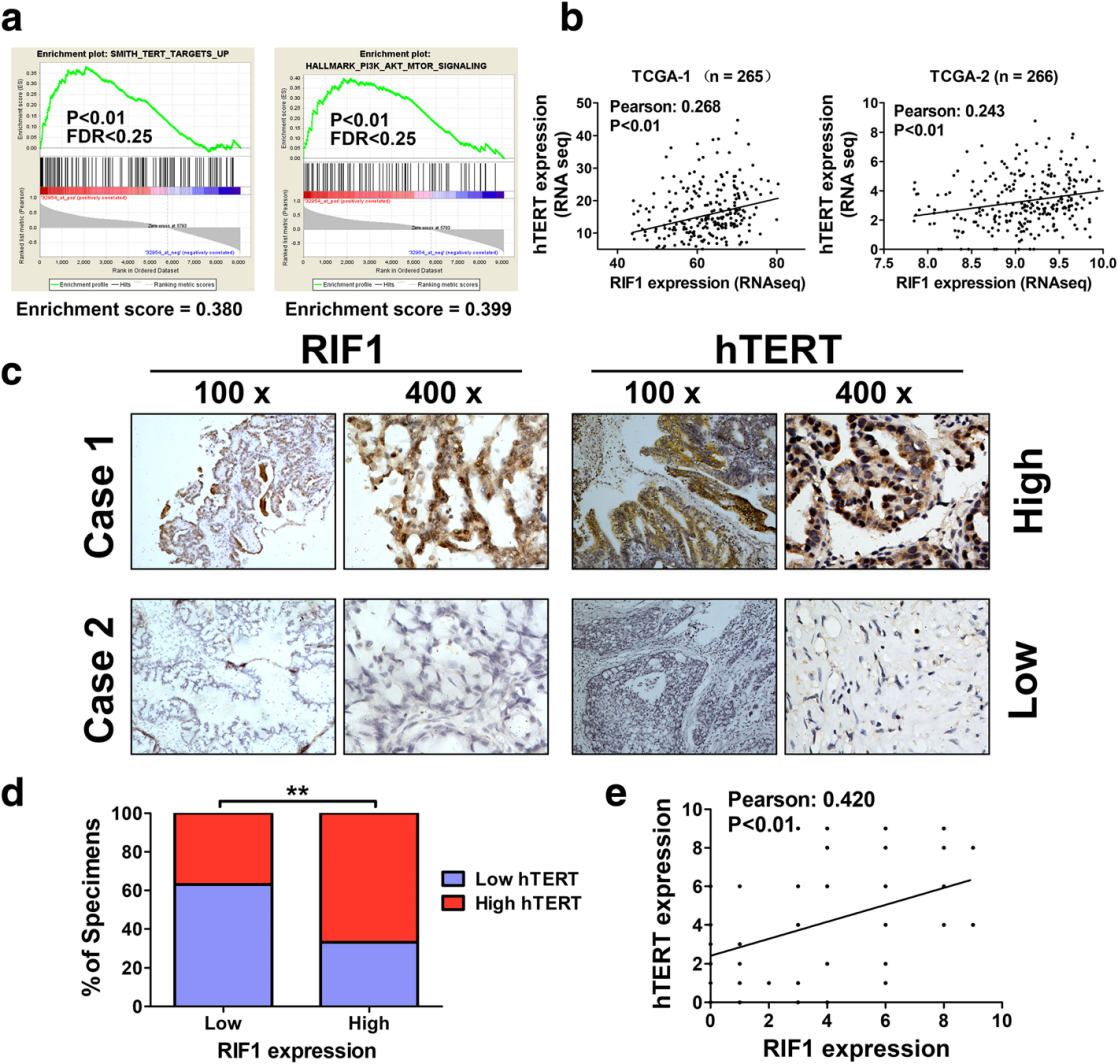
Clinical relevance of RIF1 and hTERT in EOC. **a** GSEA plot showing that RIF1 expression was positively correlated with TERT-activated gene signatures and TERT downstream PI3K/AKT signaling in the GEO data set (NCBI/GEO/GSE7463; n = 43). **b** Bioinformatics analysis based on TCGA database showed the mRNA expression levels of RIF1 positively correlated with hTERT expression level in ovarian cancer tissues. **c** and **d** Immunohistochemical staining suggesting that RIF1 expression correlated positively with hTERT expression in 75 clinical EOC specimens. Percentage of EOC specimens showing low or high RIF1 expression relative to the expression levels of hTERT. ** *P* < 0.01. **e** The relative expression of RIF1 and hTERT was used to perform the correlation analysis

Next, we investigated the potential mechanisms by which RIF1 promotes EOC cell growth and CSC-like properties. By performing Gene set enrichment analysis (GSEA) in the GEO ovarian cancer data set, we found that the RIF1 expression level was positively correlated with TERT-activated gene signatures and TERT downstream PI3K/AKT signaling (GSE7463; Fig. 4a). By analyzing the data from the publicly available TCGA database, we found that the mRNA expression levels of RIF1 positively correlated with hTERT expression level in ovarian cancer tissues (Fig. 4b). To further confirm the correlation between RIF1 and hTERT, we evaluated RIF1 and hTERT protein expression level in cancer tissue samples from 75 EOC patients via IHC. As shown in Fig. 4c, Clinical samples with high levels of RIF1 expression demonstrated high levels of hTERT expression, whereas samples with low RIF1 expression exhibited low levels of hTERT expression (Fig. 4c-d). Moreover, the protein expression levels of RIF1 positively correlated with hTERT protein expression (*r* = 0.42, *p* < 0.001) in EOC tissues (Fig. 4e).

### RIF1 binds to the promoter of hTERT and enhances hTERT promoter activity and expression in EOC cells

**Fig. 5:**
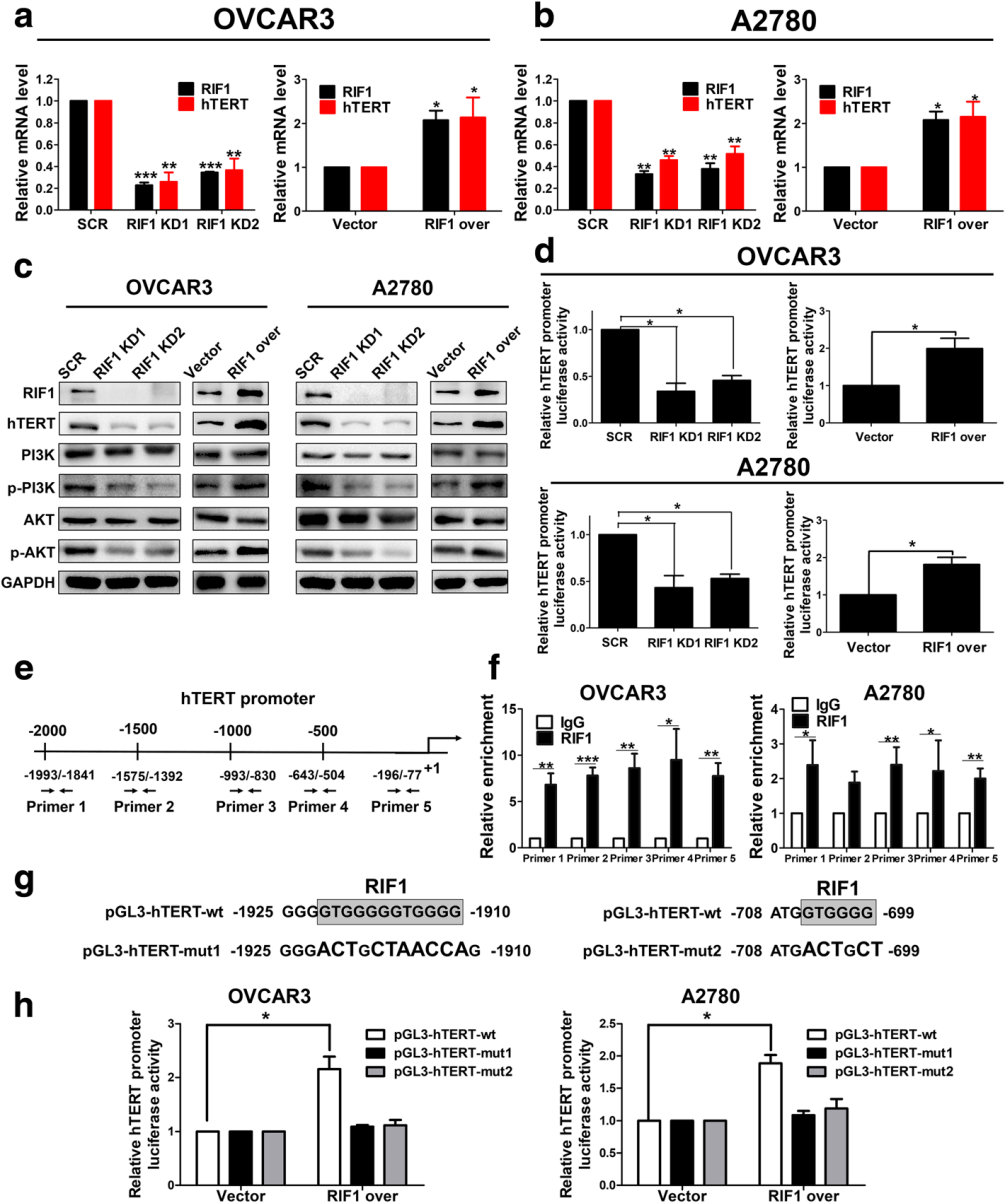
RIF1 regulated hTERT expression and promoter activity in EOC cells by binding to the promoter of *hTERT.* **a** and **b** hTERT mRNA expression was down-regulated in RIF1 knockdown and up-regulated in RIF1 overexpressed OVCAR3 and A2780 cells. **c** RIF1 regulated the protein expression of hTERT and the hTERT downstream PI3K/AKT signaling proteins in OVCAR3 and A2780 cells. **d** Relative hTERT promoter activity in RIF1 knockdown and RIF1 overexpressed OVCAR3 and A2780 cells. **e** Schematic diagram of five pairs of primers designed for ChIP spanning the *hTERT* promoter. **f** RIF1 binds to the *hTERT* promoter indicated by ChIP in OVCAR3 and A2780 cells. IgG from rabbit served as a control. **g** A schematic diagram of RIF1 wild type and mutant binding sites in hTERT promoter. **h** Dual-luciferase reporter gene assay was conducted in OVCAR3 and A2780 cells with overexpression of RIF1. Data are expressed as means ± SD of three independent experiments. * *P* < 0.05, ** *P* < 0.01, *** *P* < 0.001

Then we explored whether RIF1 can regulate hTERT mRNA and protein expression in EOC cell lines. We found that overexpression of RIF1 promoted hTERT mRNA and protein levels, while knockdown of RIF1 inhibited hTERT expression at transcriptional and translational levels in OVCAR3 and A2780 cells (Fig. 5a-c). We further checked the effect of RIF1 on the pivotal proteins in the PI3K/AKT signaling, a crucial downstream pathway associated with the hTERT signaling [28–30] and found that overexpression of RIF1 up-regulated p-PI3K and p-AKT expression, but not total PI3K and AKT in OVCAR3 and A2780. Knockdown of RIF1 had the opposite effect (Fig. 5c). These results indicate that RIF1 could promote hTERT expression and activate hTERT signaling which is involved in tumor growth and CSC-like traits.

The luciferase reporter assay suggested that overexpression of RIF1 significantly enhanced the *hTERT* promoter activity compared with the vector group in OVCAR3 and A2780 cells, whereas knockdown of RIF1 dramatically decreased the *hTERT* promoter activity in OVCAR3 and A2780 cells in comparison with the scrambled control cells (Fig. 5d).

To further validate the interaction between RIF1 and the *hTERT* promoter, we performed ChIP assay to analyze RIF1 binding to the *hTERT* promoter. Equal amounts of sonicated OVCAR3 and A2780 chromatin DNA were incubated with RIF1 antibody or IgG control. Protein A/G bead-captured chromatin DNA was amplified as a template. Five pairs of primers spanning the *hTERT* promoter were used for quantitative real-time PCR (Fig. 5e). The ChIP results indicated that RIF1 was most enriched on the promoter region from − 1993 to − 77 (Fig. 5f). Since Kanoh etc. [31] previously identified high-affinity RIF1-binding conserved motif: GTGGGGG, to further investigate that RIF1 binds to the specific site of hTERT, we constructed wild type and two mutant hTERT promoter luciferase reporter plasmids (Fig. 5g). We co-transfected RIF1 expression plasmid with wild type or mutant hTERT promoter luciferase reporter plasmid into OVCAR3 and A2780 cells, and dual-luciferase reporter gene assay indicated that RIF1 remarkably increased luciferase activity of wild type but not mutant hTERT promoter (Fig. 5h). Together, these results indicate that RIF1 binds to the promoter of *hTERT* and regulates hTERT expression at transcriptional level.

### RIF1 promotes tumor growth and stem cell-like phenotype in EOC via hTERT signaling pathway

**Fig. 6:**
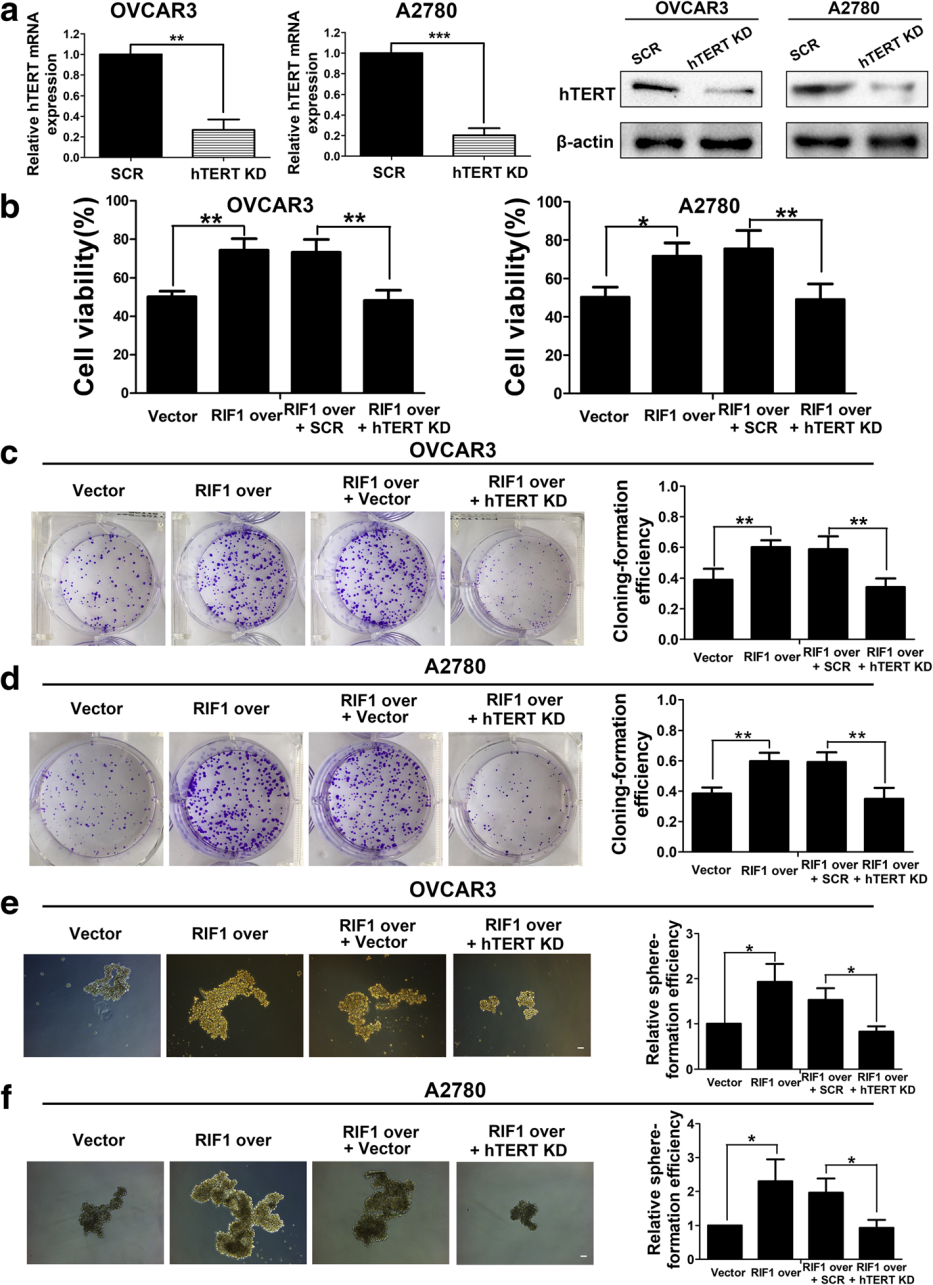
Knockdown of hTERT abrogated the promotion of cell viability, colony formation and sphere formation mediated by RIF1 overexpression in EOC cells. **a** Efficiency of hTERT knockdown (hTERT KD) in OVCAR3 and A2780 cells by shRNA was verified by real-time qPCR and western blot. **b** Cell viability at 72 h was examined by absorbance at the wavelength of 490 nm and normalized to the absorbance value in the control group by MTS assay. **c** and **d** Colony formation assay of control vector or RIF1-overexpressed OVCAR3 and A2780 cells with or without hTERT knockdown. **e** and **f** Representative images and quantification of spheres formed by control vector or RIF1-overexpressed OVCAR3 and A2780 cells with or without hTERT knockdown. Scale bar, 100 μm. The results were shown as means ± SD of three independent experiments. * *P* < 0.05, ** *P* < 0.01

**Fig. 7:**
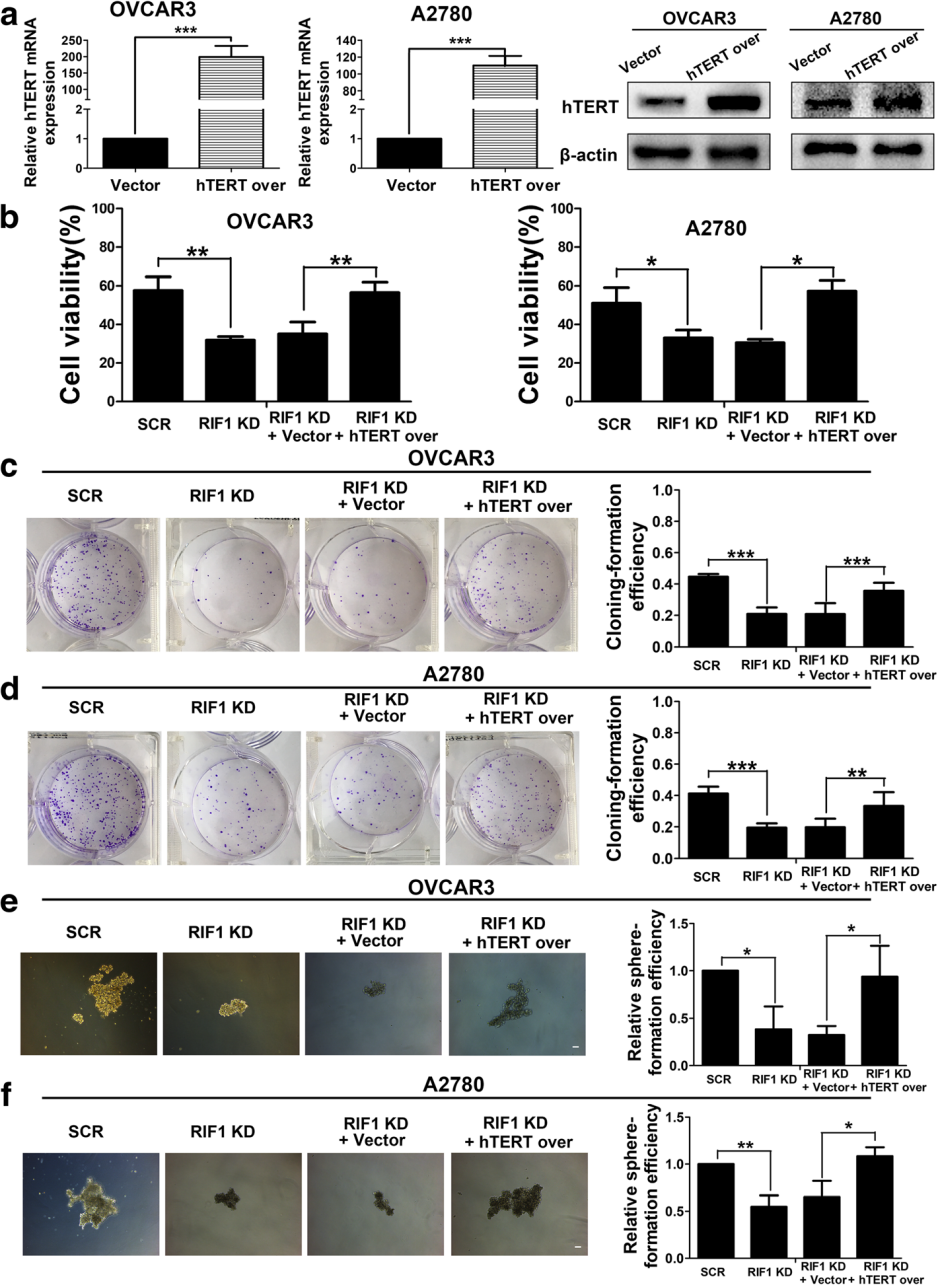
Overexpression of hTERT reversed the inhibition of cell viability, colony formation sphere formation mediated by RIF1 knockdown in EOC cells. **a** Efficiency of hTERT overexpression (hTERT over) in OVCAR3 and A2780 cells was verified by real-time qPCR and western blot. **b** Cell viability at 72 h was examined by MTS assay. **c** and **d** Colony formation assay of scrambled control or RIF1 knockdown OVCAR3 and A2780 cells with or without hTERT overexpression. **e** and **f** Representative images and quantification of spheres formed by scrambled control or RIF1 knockdown OVCAR3 and A2780 cells with or without hTERT overexpression. Scale bar, 100 μm. The results were shown as means ± SD of three independent experiments. * *P* < 0.05, ** *P* < 0.01, *** *P* < 0.001

Since hTERT signaling is considered crucial for carcinogenesis and the maintenance of cancer cellular stemness of EOC, then we explored whether RIF1 could enhance tumor growth and CSC-like traits in EOC via hTERT signaling. We first detected the efficiency of hTERT shRNA(hTERT KD)and found that the hTERT shRNA could significantly decrease the expression of hTERT in mRNA and protein level in EOC cells (Fig. 6a). Then we examined the impact of silencing hTERT on tumor growth and the CSC-like traits of RIF1-overexpressed EOC cells. We used specific hTERT shRNA in RIF1-overexpressed EOC cells. We found that inhibition of hTERT signaling reduced cell viability, colony formation and stem cell-like properties in RIF1-overexpresed EOC cells (Fig. 6b-f). Since we have found RIF1 could regulate stemness related gene expression in Fig. 3f, to further confirm whether or not this regulation is dependent on hTERT, we explored whether hTERT knockdown could rescue the effect of RIF1 overexpression. As shown in Additional file 3: Figure S1, hTERT knockdown dramatically diminished the expression of RIF1-induced stemness markers NANOG and SOX2 by Western blotting, which confirmed the stem cell-like phenotype of OVCAR3 and A2780 cells. Consistently, overexpression of hTERT could rescue the inhibition effect of RIF1 knockdown on tumor growth and sphere formation. We detected the efficiency of full-length human hTERT plasmid (hTERT over) and found that the hTERT plasmid could significantly increase the expression of hTERT in mRNA and protein level in EOC cells (Fig. 7a). Then we examined the impact of hTERT overexpression on tumor growth and the CSC-like traits of RIF1-suppressed EOC cells. We found that overexpression of hTERT increased cell viability, colony formation and CSC-like properties in RIF1-suppresed EOC cells (Fig. 7b-f). Together, these results indicate that RIF1 promotes tumor growth and stem cell-like phenotype in EOC via hTERT signaling pathway.

### Knockdown of RIF1 inhibits tumourigenicity of EOC cells in vivo

**Fig. 8:**
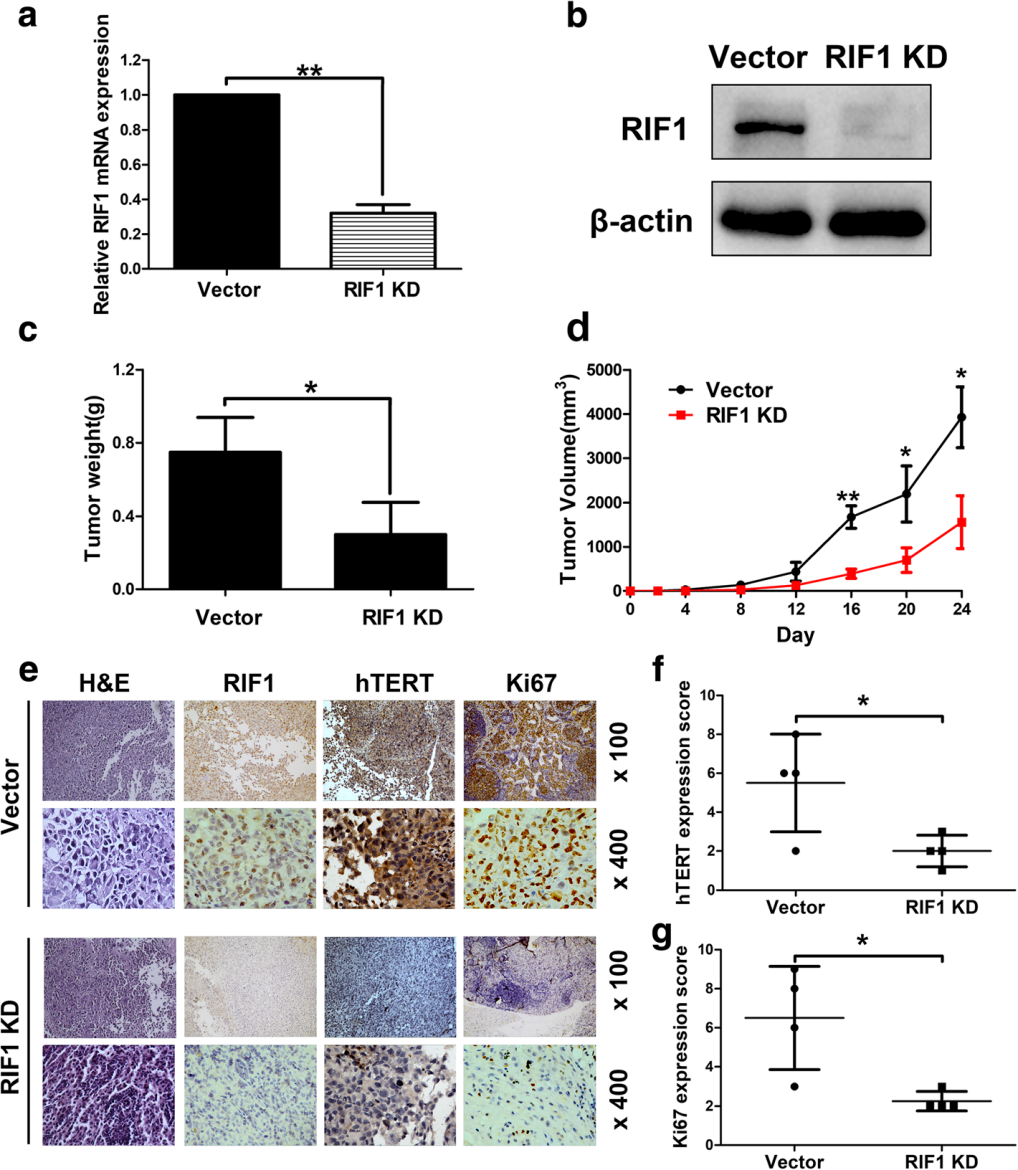
Knockdown of RIF1 inhibits tumourigenicity of EOC cells in vivo. **a** and **b** Efficiency of RIF1 stable knockdown (RIF1 KD) in OVCAR3 cells by lentivirus was verified by real-time qPCR (**a**) and western blot (**b**) after 30 generations of cell culture. **c** and **d**. 2 × 10^6^ RIF1 stable-knockdown and vector control OVCAR3 cells were inoculated subcutaneously into the right and left flanks of nude mice. Tumor weight and volume of xenografts were evaluated. **e** Representative images of H&E and IHC staining of the resected tumor. **f** and **g** IHC analysis showed that RIF1 knockdown reduced the expression of hTERT and decreased the proliferation index Ki67. Data were presented as means ± SD. * *P* < 0.05, ** *P* < 0.01

We established stable RIF1-knockdown OVCAR3 cells with lentiviral vectors. Our data showed these stable RIF1-knockdown cells had low expression of RIF1 at both mRNA and protein levels compared with the vector control cells (Vector) after 30 generations of cell culture (Fig. 8a and b). To further confirm the effect of RIF1 on the tumourigenic capacity of OVCAR3 cells in vivo, RIF1 stable-knockdown or control OVCAR3 cells were injected subcutaneously in BALB/c nude mice. On day 24, the animals were euthanized and the tumors were excised and weighed. Tumor weight in RIF1 knockdown group were greatly reduced compared to the control group (Fig. 8c). Tumor cells with RIF1 knockdown grew more slowly than the vector control in the same mouse (Fig. 8d). The tumors were resected and analyzed H&E and IHC staining. Tumor tissues derived from RIF1 knockdown cells exhibited reduced positivity for Ki67 and hTERT compared with the control groups (Fig. 8e-g), indicating that RIF1 promoted hTERT expression and EOC tumor growth in the mouse model.

## Discussion

As the rate-limiting catalytic subunit of telomerase, hTERT plays a decisive role in cell immortalization. Currently, hTERT is considered to be a hallmark of cancer and a novel target for cancer therapy, playing a pivotal role in tumorigenesis and CSC-like traits independent of its effects on telomeres [7, 8]. For example, hTERT promotes cell invasion of telomerase-negative U2OS cells [32]. Besides, Liu et al. [7] reported that hTERT stimulates EMT and induces stemness of cancer cells, thereby promoting cancer metastasis and recurrence independent of its telomere-lengthening function or telomerase activity. In the field of ovarian cancer research, previous studies have shown that hTERT is upregulated in over 95% of ovarian carcinomas and mediates ovarian cancer aggressiveness [6, 33]. For instance, Choi et al. [34] proposed that hTERT contributes to the stress hormone norepinephrine-induced ovarian cancer cell EMT, and subsequently to cell invasion and metastasis. Therefore, the mechanisms involved in hTERT regulation in EOC deserve to be fully elucidated. Previous studies demonstrated that the expression of hTERT could be regulated by chromatin remodeling or promoter methylation [35, 36] as well as by transcription factors, such as c-Myc, USF1/2, Sp1, p53 and TGF-β [4, 37, 38]. This study presents a novel mechanism involved in hTERT expression regulation in EOC, indicating that hTERT transcription activation depends on RIF1.

RIF1 has recently been demonstrated to play a pivotal role in stem cell pluripotency, DNA replication regulation and DNA repair pathway [20, 39, 40]. In the field of cancer research, RIF1 was reported upregulated in breast cancer and cervical cancer [21, 22]. In budding yeast, RIF1 is part of the telosome as RAP1 interacting factor 1 with a role in telomere maintenance [10–12]. RAP1 is a member of the shelterin complex which play a fundamental role in the protection of telomeres [41]. Interestingly, the structure and function of RIF1 are not conserved from yeasts to mammalian cells. RIF1 has Rap1 binding motif only in the budding yeast and localizes to native (capped) telomeres only in yeasts [42]. Thus, the effect of RIF1 on telomere length regulation in yeasts is not conserved in mammalian cells and particularly in human cancer cells. Actually, the relationship between human RIF1 and hTERT as well as the role of RIF1 in ovarian cancer progression is still unknown.

Our results indicated that RIF1 increased hTERT expression and then activated hTERT downstream PI3K/AKT signaling. Furthermore, RIF1 activated *hTERT* transcription and promoted *hTERT* promoter activity. Then we used CHIP assay and luciferase reporter gene assay to further verify that RIF1 specifically bound to the *hTERT* promoter (Fig. 5). Besides, Gene set enrichment analysis (GSEA) revealed that RIF1 expression closely correlated with the expression of a set of stemness regulating genes in the publicly available ovarian patient expression profiles. CSCs play a significant role in tumorigenesis, and cancer recurrence [12, 43]. In various human cancer types, including ovarian cancer, preclinical data indicates that hTERT signaling contributes to the maintenance of the CSC population [7, 33]. Consistent with the results of gene set enrichment analysis, knockdown of RIF1 inhibited EOC cell growth and CSC-like traits. (Figs. 2 and 3). RIF1 knockdown also inhibited tumorigenesis in xenograft mouse model (Fig. 4). The rescue experiments suggested that on the one hand, overexpression of hTERT rescued the inhibition of EOC cell growth and CSS-like traits mediated by RIF1 knockdown. On the other hand, knockdown of hTERT abrogated the promotion of cell growth, colony formation and CSS-like traits mediated by RIF1 overexpression in EOC cells. Analysis of clinical samples in this study demonstrated that RIF1 expression was frequently up-regulated in EOC tissues and EOC cell lines compared with normal ovarian tissues and normal ovarian epithelial cell line and its overexpression associated with FIGO Stage (*P* < 0.001), overall survival (*P* = 0.0012) and progression-free survival (P < 0.001) of ovarian cancer patients. Moreover, the expression of RIF1 is positively correlated with hTERT expression in ovarian cancer tissues in two separate gene expression profiles from TCGA database and our own EOC tissue samples. All these results indicate that RIF1 regulates EOC tumor growth and progression through binding to the promoter of *hTERT* and regulating hTERT expression.

## Conclusions

In summary, our study has demonstrated that as an oncogene, RIF1 facilitates tumor cell growth and CSC-like traits in EOC. Furthermore, we have found that RIF1 exerts its oncogenic effect through the up-regulation of hTERT in EOC. Our study has provided new insights for the understanding of the regulation of hTERT in EOC tumorigenesis, revealing that RIF1 is a pivotal regulatory molecule in the hTERT signaling pathway for tumor progression as well as a potentially effective target for EOC therapy.

## Additional files

Additional file 1: **Table S1**. Sequences of primers for quantitative real-time PCR. (DOCX 13 kb)

Additional file 2: **Table S2**. Correlation between the RIF1 expression and the clinical features of ovarian cancer patients. (DOCX 14 kb)

Additional file 3: **Figure S1**. The expression of stemness related genes NANOG and SOX2 was assessed by Western blotting in OVCAR3 and A2780 cells. (TIF 819 kb)

## Abbreviations

CHIP: Chromatin immunoprecipitation
CSCs: Cancer stem cells
DSB: DNA double-strand break
EOC: Epithelial ovarian cancer
ESCs: Embryonic stem cells
GSEA: Gene set enrichment analysis
hTERT: Human telomerase reverse transcriptase
IHC: Immunohistochemistry
RIF1: Replication timing regulatory factor 1

## References

[1] Siegel RL, Miller KD, Jemal A (2018). Cancer statistics, 2018. CA Cancer J Clin.

[2] Chen L, Yao Y, Sun L, Zhou J, Miao M, Luo S (2017). Snail driving alternative splicing of CD44 by ESRP1 enhances invasion and migration in epithelial ovarian Cancer. Cell Physiol Biochem.

[3] Xie X, Yang M, Ding Y, Yu L, Chen J (2017). Formyl peptide receptor 2 expression predicts poor prognosis and promotes invasion and metastasis in epithelial ovarian cancer. Oncol Rep.

[4] Zhang N, Zhang R, Zou K, Yu W, Guo W, Gao Y (2017). Keratin 23 promotes telomerase reverse transcriptase expression and human colorectal cancer growth. Cell Death Dis.

[5] Blackburn EH (1991). Structure and function of telomeres. Nature.

[6] Shay JW, Bacchetti S (1997). A survey of telomerase activity in human cancer. Eur J Cancer.

[7] Liu Z, Li Q, Li K, Chen L, Li W, Hou M (2013). Telomerase reverse transcriptase promotes epithelial-mesenchymal transition and stem cell-like traits in cancer cells. Oncogene.

[8] Low KC, Tergaonkar V (2013). Telomerase: central regulator of all of the hallmarks of cancer. Trends Biochem Sci.

[9] Xie M, Chen Q, He S, Li B, Hu C (2011). Silencing of the human TERT gene by RNAi inhibits A549 lung adenocarcinoma cell growth in vitro and in vivo. Oncol Rep.

[10] Kubo T, Takigawa N, Osawa M, Harada D, Ninomiya T, Ochi N (2013). Subpopulation of small-cell lung cancer cells expressing CD133 and CD87 show resistance to chemotherapy. Cancer Sci.

[11] Bertolini G, Roz L, Perego P, Tortoreto M, Fontanella E, Gatti L (2009). Highly tumorigenic lung cancer CD133+ cells display stem-like features and are spared by cisplatin treatment. Proc Natl Acad Sci U S A.

[12] Huang BS, Luo QZ, Han Y, Huang D, Tang QP, Wu LX (2017). MiR-223/PAX6 Axis regulates glioblastoma stem cell proliferation and the chemo resistance to TMZ via regulating PI3K/Akt pathway. J Cell Biochem.

[13] Hardy CF, Sussel L, Shore D (1992). A RAP1-interacting protein involved in transcriptional silencing and telomere length regulation. Genes Dev.

[14] Hayano M, Kanoh Y, Matsumoto S, Renard-Guillet C, Shirahige K, Masai H (2012). Rif1 is a global regulator of timing of replication origin firing in fission yeast. Genes Dev.

[15] Yamazaki S, Ishii A, Kanoh Y, Oda M, Nishito Y, Masai H (2012). Rif1 regulates the replication timing domains on the human genome. EMBO J.

[16] Alver RC, Chadha GS, Gillespie PJ, Blow JJ. Reversal of DDK-Mediated MCM Phosphorylation by Rif1-PP1 Regulates Replication Initiation and Replisome Stability Independently of ATR/Chk1. Cell Rep. 2017;18:2508-20. 10.1016/j.celrep.2017.02.042 PMC5357733 28273463

[17] Foti R, Gnan S, Cornacchia D, Dileep V, Bulut-Karslioglu A, Diehl S (2016). Nuclear architecture organized by Rif1 underpins the replication-timing program. Mol Cell.

[18] Adams IR, McLaren A (2004). Identification and characterisation of mRif1: a mouse telomere-associated protein highly expressed in germ cells and embryo-derived pluripotent stem cells. Dev Dyn.

[19] Dan J, Liu Y, Liu N, Chiourea M, Okuka M, Wu T, et al. Rif1 maintains telomere length homeostasis of ESCs by mediating heterochromatin silencing. Dev Cell. 2014;29:7-19. 10.1016/j.devcel.2014.03.004 PMC4720134 24735877

[20] Wang J, Rao S, Chu J, Shen X, Levasseur DN, Theunissen TW (2006). A protein interaction network for pluripotency of embryonic stem cells. Nature.

[21] Mei Y, Peng C, Liu YB, Wang J, Zhou HH (2017). Silencing RIF1 decreases cell growth, migration and increases cisplatin sensitivity of human cervical cancer cells. Oncotarget.

[22] Wang H, Zhao A, Chen L, Zhong X, Liao J, Gao M (2009). Human RIF1 encodes an anti-apoptotic factor required for DNA repair. Carcinogenesis.

[23] Zeng ZL, Luo HY, Yang J, Wu WJ, Chen DL, Huang P (2014). Overexpression of the circadian clock gene Bmal1 increases sensitivity to oxaliplatin in colorectal cancer. Clin Cancer Res.

[24] Samaeekia R, Adorno-Cruz V, Bockhorn J, Chang YF, Huang S, Prat A (2017). miR-206 inhibits Stemness and metastasis of breast Cancer by targeting MKL1/IL11 pathway. Clin Cancer Res.

[25] Chang YW, Chiu CF, Lee KY, Hong CC, Wang YY, Cheng CC (2015). CARMA3 represses metastasis suppressor NME2 to promote lung Cancer Stemness and metastasis. Am J Respir Crit Care Med.

[26] Loh YH, Wu Q, Chew JL, Vega VB, Zhang W, Chen X (2006). The Oct4 and Nanog transcription network regulates pluripotency in mouse embryonic stem cells. Nat Genet.

[27] Basu-Roy U, Seo E, Ramanathapuram L, Rapp TB, Perry JA, Orkin SH (2012). Sox2 maintains self renewal of tumor-initiating cells in osteosarcomas. Oncogene.

[28] Liu T, Li W, Lu W, Chen M, Luo M, Zhang C (2017). RBFOX3 promotes tumor growth and progression via hTERT signaling and predicts a poor prognosis in hepatocellular carcinoma. Theranostics.

[29] Zhao Q, Wang XY, Yu XX, Zhai YX, He X, Wu S (2015). Expression of human telomerase reverse transcriptase mediates the senescence of mesenchymal stem cells through the PI3K/AKT signaling pathway. Int J Mol Med.

[30] Shi YA, Zhao Q, Zhang LH, Du W, Wang XY, He X (2014). Knockdown of hTERT by siRNA inhibits cervical cancer cell growth in vitro and in vivo. Int J Oncol.

[31] Kanoh Y, Matsumoto S, Fukatsu R, Kakusho N, Kono N, Renard-Guillet C (2015). Rif1 binds to G quadruplexes and suppresses replication over long distances. Nat Struct Mol Biol.

[32] Yu ST, Chen L, Wang HJ, Tang XD, Fang DC, Yang SM (2009). hTERT promotes the invasion of telomerase-negative tumor cells in vitro. Int J Oncol.

[33] Meng E, Taylor B, Ray A, Shevde LA, Rocconi RP (2012). Targeted inhibition of telomerase activity combined with chemotherapy demonstrates synergy in eliminating ovarian cancer spheroid-forming cells. Gynecol Oncol.

[34] Choi MJ, Cho KH, Lee S, Bae YJ, Jeong KJ, Rha SY (2015). hTERT mediates norepinephrine-induced slug expression and ovarian cancer aggressiveness. Oncogene.

[35] Shin KH, Kang MK, Dicterow E, Park NH (2003). Hypermethylation of the hTERT promoter inhibits the expression of telomerase activity in normal oral fibroblasts and senescent normal oral keratinocytes. Br J Cancer.

[36] Kyo S, Takakura M, Fujiwara T, Inoue M (2008). Understanding and exploiting hTERT promoter regulation for diagnosis and treatment of human cancers. Cancer Sci.

[37] Goueli BS, Janknecht R (2003). Regulation of telomerase reverse transcriptase gene activity by upstream stimulatory factor. Oncogene.

[38] Renaud S, Loukinov D, Bosman FT, Lobanenkov V, Benhattar J (2005). CTCF binds the proximal exonic region of hTERT and inhibits its transcription. Nucleic Acids Res.

[39] Zimmermann M, Lottersberger F, Buonomo SB, Sfeir A, de Lange T (2013). 53BP1 regulates DSB repair using Rif1 to control 5′ end resection. Science.

[40] Hiraga SI, Ly T, Garzon J, Horejsi Z, Ohkubo YN, Endo A, et al. Human RIF1 and protein phosphatase 1 stimulate DNA replication origin licensing but suppress origin activation. EMBO Rep. 2017; 10.15252/embr.201641983 PMC5331243 28077461

[41] Mattarocci S, Reinert JK, Bunker RD, Fontana GA, Shi T, Klein D (2017). Rif1 maintains telomeres and mediates DNA repair by encasing DNA ends. Nat Struct Mol Biol.

[42] Mattarocci S, Hafner L, Lezaja A, Shyian M, Shore D (2016). Rif1: a conserved regulator of DNA replication and repair hijacked by telomeres in yeasts. Front Genet.

[43] Koury J, Zhong L, Hao J (2017). Targeting signaling pathways in Cancer stem cells for Cancer treatment. Stem Cells Int.

