# Supplementary material for: RIF1 promotes human epithelial ovarian cancer growth and progression via activating human telomerase reverse transcriptase expression
Source: J Exp Clin Cancer Res. 2018 Aug 3;37:182. doi: 10.1186/s13046-018-0854-8 (PMC6091081; doi:10.1186/s13046-018-0854-8)
Supplement: Supplementary file 1 — Table S1. Sequences of primers for quantitative real-time PCR. (DOCX 13 kb) [file 13046_2018_854_MOESM1_ESM.docx]

| **Table S1 Sequences of primers for quantitative real-time PCR** | | |
| --- | --- | --- |
| Gene name | Forward primer sequence (5' → 3') | Reverse primer sequence (5' → 3') |
| β-actin | CATGTACGTTGCTATCCAGGC | CTCCTTAATGTCACGCACGAT |
| RIF1 | TGGCAGATGACATTGATAGA | TAGATTGTGTAGTAGGAGAAGTT |
| hTERT | AAATGCGGCCCCTGTTTCT | CAGTGCGTCTTGAGGAGCA |
