# Supplementary material for: RIF1 promotes human epithelial ovarian cancer growth and progression via activating human telomerase reverse transcriptase expression
Source: J Exp Clin Cancer Res. 2018 Aug 3;37:182. doi: 10.1186/s13046-018-0854-8 (PMC6091081; doi:10.1186/s13046-018-0854-8)
Supplement: Supplementary file 2 — Table S2. Correlation between the RIF1 expression and the clinical features of ovarian cancer patients. (DOCX 14 kb) [file 13046_2018_854_MOESM2_ESM.docx]

**Table S2 Correlation between the RIF1 expression and the clinical features of ovarian cancer patients.**

| **Clinical features** | **n** | **RIF1 level** | | **χ^2^** | **P** |
| --- | --- | --- | --- | --- | --- |
|  |  | **Low** | **High** |  |  |
| **Age** |  | | | 0.324 | 0.569 |
| ≥50 | 34 | 16 | 18 |  |  |
| <50 | 41 | 22 | 19 |  |  |
| **Histological type** |  | | | 2.996 | 0.083 |
| Serous | 37 | 15 | 22 |  |  |
| No serous | 38 | 23 | 15 |  |  |
| **Differentiation** |  | | | 0.641 | 0.623 |
| Low | 31 | 14 | 17 |  |  |
| Middle and High | 44 | 24 | 20 |  |  |
| **Stage** |  | | | 10.277 | **0.001^*^** |
| I/II | 30 | 22 | 8 |  |  |
| III/IV | 45 | 16 | 29 |  |  |

**^*^p < 0.05 was considered significant.**
