# Supplementary figures and images for: RIF1 promotes human epithelial ovarian cancer growth and progression via activating human telomerase reverse transcriptase expression
Source: J Exp Clin Cancer Res. 2018 Aug 3;37:182. doi: 10.1186/s13046-018-0854-8 (PMC6091081; doi:10.1186/s13046-018-0854-8)

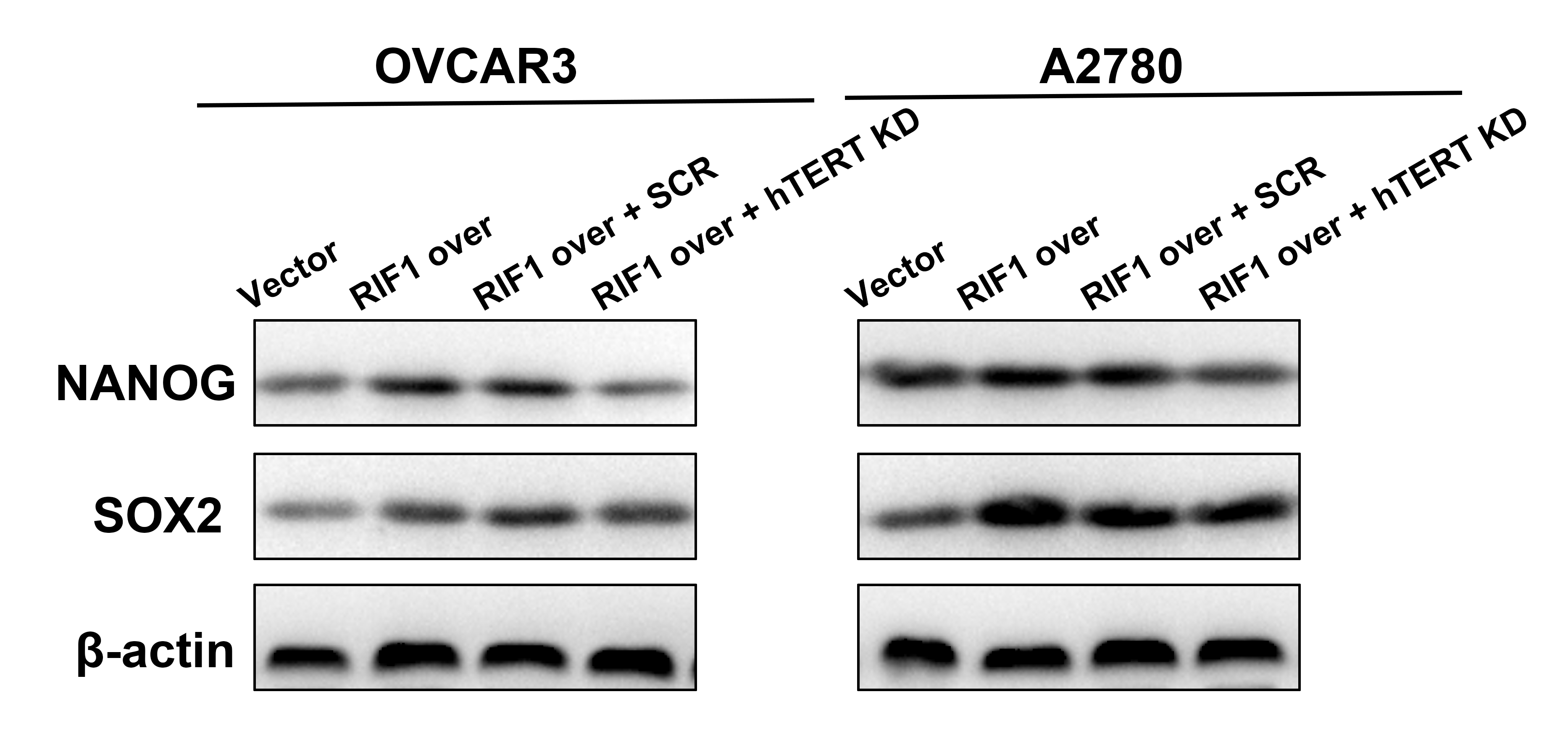

Supplement: Supplementary file 3 — Figure S1. The expression of stemness related genes NANOG and SOX2 was assessed by Western blotting in OVCAR3 and A2780 cells. (TIF 819 kb) [file 13046_2018_854_MOESM3_ESM.tif]
